# A Novel Support Vector Machine with Globality-Locality Preserving

**DOI:** 10.1155/2014/872697

**Published:** 2014-06-17

**Authors:** Cheng-Long Ma, Yu-Bo Yuan

**Affiliations:** Institute of Metrology and Computational Science, China Jiliang University, Hangzhou, Zhejiang 310018, China

## Abstract

Support vector machine (SVM) is regarded as a powerful method for pattern classification. However, the solution of the primal optimal model of SVM is susceptible for class distribution and may result in a nonrobust solution. In order to overcome this shortcoming, an improved model, support vector machine with globality-locality preserving (GLPSVM), is proposed. It introduces globality-locality preserving into the standard SVM, which can preserve the manifold structure of the data space. We complete rich experiments on the UCI machine learning data sets. The results validate the effectiveness of the proposed model, especially on the Wine and Iris databases; the recognition rate is above 97% and outperforms all the algorithms that were developed from SVM.

## 1. Introduction

In the past decades, support vector machine (SVM) [1] was thought to be a powerful tool for classification tasks. It can separate different classes by hyperplanes, which are determined by optimal directions and support vectors, while the optimal directions are obtained by maximizing the margins between each two classes. SVM and its variants [2–5] have been successfully applied to many research areas such as face detection and recognition [6], speech recognition [7], text classification [8], and image retrieval [9].

It is well known that SVM is an optimization problem. The optimal solution can be found by solving a quadratic programming problem. Because the objective function is convex, the global minimum solution is guaranteed. However, the traditional SVM solution is susceptible to class distribution, which means it is nonrobust to data samples. So, in order to overcome this shortcoming, Zafeiriou et al. [10] proposed a minimum class variance support vector machine (MCVSVM), which was inspired by optimization Fisher's discriminant ratio. By taking the manifold structure of the data space into consideration, Wang et al. [11] introduced within-class locality preserving into SVM and proposed the minimum class locality preserving variance support vector machine (MCLPV_SVM). Besides, since SVM deals with a subset of data points (support vectors) rather than the entire data set, so to some extent, SVM solution is based on “local" characteristics of the data; therefore, Xiong and Cherkassky [12] incorporated global discriminant information into SVM and proposed the SVM+LDA. Analogously, Khan et al. [13] presented a novel SVM+NDA (nonparametric discriminant analysis) model for classification; it fused some partially global information and local information. In particular, both SVM+LDA and SVM+NDA can cope with the small sample problem, which benefited from the construction method of the models.

According to the above analysis, none of the mentioned methods takes the manifold structure of the data space into consideration, except the MCLPV_SVM method, but MCLPV_SVM loses some discriminant information. In the basic learning algorithms, the locality of learning data set should be considered; in recent years, many excellent publications showed the importance of locality, such as [14–16]. It is also important for finding the clusters in high dimensional data set, such as [17, 18]. Recently, discriminant locality preserving projections (DLPP) [19–21] is proposed, and it is keen to find the subspace which can best discriminate different classes by maximizing the locality preserving between-class distances while minimizing the locality preserving within-class distances. So, DLPP can not only preserve local structure, but also implicate discriminant information. Inspired by DLPP, and considering that the mean sample can reflect the characteristics of data structure, which is also center-invariant in each class [22], this paper proposed a novel learning algorithm, called support vector machine with globality-locality preserving (GLPSVM). It introduced globality and locality preserving ability into the SVM. The proposed method preserves intrinsic manifold structure of the data space, takes the class distribution into consideration, and obtains a robust solution.

In summary, this paper is organized as follows.  Section 2 gives a brief review of SVM.  Section 3 gives the proposed method, including derivation, solving, and analysis. The experimental results are given in Section 4. Finally, conclusions are in Section 5.

## 2. A Brief Review of SVM

Given a set of pairwise samples **S** = {(**x**_*i*_,*y*_*i*_)}_*i*=1_^*n*^, where **x**_*i*_ ∈ *R* ^*m*^ is a sample point in *m*-dimensional space and *y*_*i*_ ∈ {+1, −1} is the corresponding label. The direct way to separate these samples into two classes is to find a separating hyperplane.

For the linearly separable case, the SVM model is as follows:

(1) min⁡w 12||w||2s.t   yi(wTxi+b)≥1, ∀i=1,…,n.

By transforming this optimization problem into its corresponding dual problem, the optimal discriminant vectors can be found through

(2) $$\begin{array}{c} w=\overset{n}{\underset{i=1}{\sum }}\alpha_{i}y_{i}x_{i}, \end{array}$$

where *α*_*i*_ and **x**_*i*_ are the dual variable and data sample (called support vector), respectively. The support vectors are crucial for classification since removing these points may change the solution of SVM. In SVM, the separable directions are decided by the optimal discriminant vectors obtained through (2). So, if we project the data into a feature space spanning by the optimal discriminant vectors, then these data will be separable in the feature space.

Usually, in real world applications, we need to deal with the multiclassification cases, such as face recognition [6] and text categorization [8]. In such cases, we need to extend SVM to multiclass SVM. The general approach is to code the classes according to a certain strategy, like one-against-all (OAA) or one-against-one (OAO) [23]. The OAA coding approach compares data in a single class with all the samples in others classes to generate the decision boundary; in this case, *c*-many decision boundaries are built for *c*-many classes. The OAO strategy generates decision boundaries from all possible pair of classes, which obtains *c*(*c* − 1)/2-many decision boundaries. Comparatively, the OAO can obtain more discriminant vectors than OAA but will cost more computational time.

## 3. The Proposed GLPSVM

In this section, we will propose a novel support vector classier which takes the class distribution into consideration, and a robust solution is expected. Firstly, we will introduce the definition of globality-locality preserving.

### 3.1. Globality-Locality Preserving (GLP)

Discriminant locality preserving projections (DLPP) [19–21] is a powerful method for extracting the manifold structure of data samples. Given a set of samples **X** = {**x**_*i*_}_*i*=1_^*n*^ ∈ *R* ^*m*×*n*^, where **x**_*i*_ is a sample point in *m*-dimensional space, and *Y* = {*y*_*i*_}_*i*=1_^*n*^ ∈ *R* ^1×*n*^ is the corresponding labels. Let *y*_*i*_ ∈ {+1, −1} and *C* = {*C*_+_, *C*_−_}, and then all *y*_*i*_ = +1 label samples belonging to *C*_+_ and the others belonging to *C*_−_. Thus, we have *n* = *n*_1_ + *n*_2_, where *n*_1_ is the number of samples in *C*_+_ and *n*_2_ is the number of samples in *C*_−_. Suppose that **T** = {**t**_*i*_}_*i*=1_^*n*^ ∈ *R* ^*d*×*n*^ is the low-dimensional feature projections of **X** and DLPP tries to maximize an objective function as follows:

(3) $$\begin{array}{c} J=\frac{\sum_{i,j=1}^{2}\left(m_{i}-m_{j}\right)B_{ij}{\left(m_{i}-m_{j}\right)}^{T}}{\sum_{s=1}^{2}\sum_{i,j=1}^{n_{s}}\left(t_{i}^{s}-t_{j}^{s}\right)W_{ij}^{s}{\left(t_{i}^{s}-t_{j}^{s}\right)}^{T}}, \end{array}$$

where **m**_*i*_ and **m**_*j*_ represent the mean vectors of the projected samples in the *i*th and *j*th class, respectively. *W*_*ij*_^*s*^ and *B*_*ij*_ are elements of the within-class weight matrix **W** and the between-class weight matrix **B** defined as

(4) Wijs={exp⁡(−||xi−xj||2σ1),xi∈Nk1(xj)  or  xj∈Nk1(xi)0otherwise,Bij={exp⁡(−||ui−uj||2σ2),ui∈Nk2(uj)  or  uj∈Nk2(ui)0otherwise,

where *N*_*k*_1__(**x**), *N*_*k*_2__(**u**) denote local neighbors of **x** and **u**, respectively, *k*_1_ is the sample neighborhood, and *k*_2_ is the mean sample neighborhood. The parameters *σ*_1_ and *σ*_2_ are empirically determined, and **u**_*i*_ is the mean vector of samples in the *i*th class. Suppose **w** : **X** → **T** is a mapping from high-dimensional data space to low-dimensional feature space; that is, **T** = **w** ^*T*^ **X**. Then, the objection function (3) can be rewritten as follows:

(5) $$\begin{array}{c} J\left(w\right)=\frac{w^{T}UHU^{T}w}{w^{T}XLX^{T}w}, \end{array}$$

where **U** = [**u**_1_, **u**_2_], **H** and **L** are the Laplacian matrices [24, 25], **H** = **D**_*b*_ − **B**, **B** = [*B*_*ij*_]_*i*,*j*=1_^2^, **D**_*b*_ is a diagonal matrix and its elements are column (or row) sums of **B**, **L** = **D**_*w*_ − **W**, **W** is a block diagonal matrix, that is, **W** = diag⁡{[*W*_*ij*_^*s*^]_*i*,*j*=1_^*n*_*s*_^}_*s*=1_^2^, and **D**_**w**_ is also a block diagonal matrix; each block of **D**_**w**_ is a diagonal matrix and its elements are the column (or row) sums of each block of **W**.

Formula (5) is also called locality preserving discriminant ratio (criterion); that is, DLPP is keen to find the feature subspace via maximizing this ratio, which means simultaneously maximizing the locality preserving between-class distance and minimizing the locality preserving within-class distance.

On the other hand, Huang et al. [22] had another view of the weight matrix of DLPP, and they believe that the mean sample is center-invariant in the same class, and it can reflect the characteristics of data structure, so to some extent it decides the accuracy in classification tasks. In this paper, we preserve the structure information of mean samples, which can to a large extent make up the loss of global information when only local structure is preserved. In a word, we define locality preserving matrix and globality preserving matrix as follows:

- locality preserving matrix: **Z**_*w*_ = **X** **L** **X** ^*T*^,
- globality preserving matrix: **Z**_*b*_ = **U** **H** **U** ^*T*^.

### 3.2. Derivation of GLPSVM

Now, we give the proposed extension of SVM, called GLPSVM. For the linearly separable data, GLPSVM can be described as follows:

(6) min⁡w 12wT(λZw+Zb+βI)ws.t. yi(wTxi+b)≥1, ∀i=1,…,n,

where *β* **I** represents the regularization matrix, which is added to cope with small sample problems. This model not only maximizes the margin of the separating hyperplane, but also minimizes the scatter of the data in discriminant directions, which benefited from taking both the locally manifold structure of the data space and globality manifold structure of the mean sample space into consideration. Here, *λ* is an empirically determined key parameter which controls the tradeoff.

According to the model, we can see that the optimal discriminant directions are no longer the same as classical SVM. It is because that we introduce the obtained GLP to the optimization model of SVM. The classification performance of the proposed method will be shown in Section 4.

### 3.3. Solution to the GLPSVM

Similar to SVM, the proposed model can be viewed as a quadratic optimization problem. Lagrange's method of undetermined multipliers is used to solve this problem. Suppose *α*_*i*_ (*i* = 1,2,…, *n*) is positive Lagrange multipliers, and let Δ≜*λ* **Z**_*w*_ + **Z**_*b*_ + *β* **I**, and then the corresponding Lagrangian is

(7) $$\begin{array}{c} L\left(w,b,\alpha \right)=\frac{1}{2}w^{T}\Delta w-\overset{n}{\underset{i=1}{\sum }}\alpha_{i}\left[y_{i}\left(w^{T}x_{i}+b\right)-1\right]. \end{array}$$

Taking derivatives with respect to **w** and *b*, respectively, we obtain

(8) $$\begin{array}{c} w=\Delta^{-1}\overset{n}{\underset{i=1}{\sum }}y_{i}\alpha_{i}x_{i},\\ \overset{n}{\underset{i=1}{\sum }}y_{i}\alpha_{i}=0. \end{array}$$

Hence, we have the following dual problem:

(9) max⁡ ∑i=1nαi−12∑i,j=1nyiyjαiαjxiTΔ−1xjs.t. ∑i=1nyiαi=0, αi≥0,  i=1,…,n.

Suppose α* is the optimization solution of this dual problem. Then, the optimal discriminant vectors w* can be found as

(10) $$\begin{array}{c} w^{*}=\overset{n}{\underset{i=1}{\sum }}y_{i}\alpha_{i}^{*}\Delta^{-1}x_{i}. \end{array}$$

So, the corresponding decision surface is

(11) g(x)=sign⁡(w∗Tx+b)=sign⁡(∑i=1nyiαi∗xiTΔ−1x+b∗).

Finally, the corresponding optimal bias b* can be calculated as

(12) $$\begin{array}{c} b^{*}=\frac{1}{n_{\text{sv}}}\overset{n_{\text{ sv }}}{\underset{i=1}{\sum }}\left(y_{i}-\overset{n}{\underset{j=1}{\sum }}y_{j}\alpha_{j}^{*}x_{j}^{T}\Delta^{-1}x_{i}\right), \end{array}$$

where *n*_sv_ is the number of support vectors.

As can be seen, in linearly separable case, GLPSVM is required to obtain a completely accurate decision hyperplane. However, in real world applications, the decision hyperplane no longer needs to be completely accurate, so we extend the GLPSVM to soft margin situations.

### 3.4. Soft Margin GLPSVM

Reference [13] proposed the soft margin method for SVM, to cope with cases when we do not need to obtain a completely accurate decision hyperplane; that is, we permit an error tolerability within limits. Then, the soft margin GLPSVM can be described as follows:

(13) min⁡w 12wT(λZw+Zb+βI)w+C∑i=1nξis.t. yi(wTxi+b)≥1−ξi, ξi≥0,  ∀i=1,…,n,

where *C* is a predefined positive real number and larger values of *C* correspond to higher penalty assigned to errors. *ξ* = [*ξ*_1_, *ξ*_2_,…, *ξ*_*n*_] is slack variables which reflect the degree of misclassification. Apparently, the soft margin GLPSVM is also a quadratic optimization problem, and we can solve it in the same way as standard GLPSVM.

### 3.5. Effective Algorithm for GLPSVM

Note that the objection function of classical SVM is (1/2)**w** ^*T*^ **w**; however, in this paper, we use (1/2)**w** ^*T*^Δ**w** for replacement. In fact, if we define some relational expressions as follows:

(14) $$\begin{array}{c} w^{r}=\Delta^{1/2}w,\\ x_{i}^{r}=\Delta^{-1/2}x_{i},\;\forall i=1,2,\ldots ,n, \end{array}$$

and then the GLPSVM model is equivalent to

(15) min⁡wr 12||wr||2s.t yi(wrTxir+b)≥1, ∀i=1,…,n.

Then, we can find that the solution to GLPSVM can be solved by standard SVM software package, but the optimal discriminant vectors are different. Since we introduce globality-locality preserving to SVM, the optimal discriminant directions in GLPSVM can preserve the intrinsic manifold structure of the data in low-dimensional feature space. Besides, matrices Δ^1/2^ and Δ^−1/2^ can be calculated through the eigenvalue decomposition of the matrix Δ; the interested readers can refer to literature [13] for more details.

## 4. Performance Evaluation

### 4.1. The Parameters' Influence on the Performance

In the proposed model, there are totally six parameters; they are the neighborhood parameters *k*_1_, *k*_2_ and the heat kernel parameters *σ*_1_, *σ*_2_ in locality preserving matrix and globality preserving matrix, respectively, and a trade-off parameter *λ* along with the regularization parameter *C*. In this section, to show the parameters' influence on the performance, we do experiments on a binary ionosphere database. We select 30% of samples of each class for training, the rest of samples are for testing, and all the samples are normalized before the experiment.

For the parameters setting, the regularization parameter *C* is selected from the set {0.1,1, 10,100}, the heat kernel parameter *σ* is selected from the set {0.5,1, 1.5,2, 2.5}, the neighborhood parameter *k*_1_ is selected from the set {10,20,30,40,50,60,70,80}, and the trade-off parameter *λ* is set to {0.2,0.4,0.6,0.8,1, 1.2,2}.

Firstly, we set the trade-off parameter to be 0.2 and use the same heat kernel parameter (*σ*_1_ = *σ*_2_ = *σ*) to see the effect of the parameters *C* and *k*_1_.  Table 1 shows classification accuracy under different settings of these three parameters. We can find that the regularization parameter *C* plays an important role in classification performance. Besides, appropriate parameter selection can provide better classification results.

Next, we will explore the effect of the trade-off parameter *λ*.  Table 2 presents the classification accuracy of GLPSVM under different trade-off parameter *λ* and different regularization parameter *C*. Here, we give the highest accuracy with its corresponding sample neighborhood parameter *k*_1_. It can be seen that the *λ* also plays an important role in classification results and small *λ* may be more appropriate than large *λ*.

### 4.2. Comparative Analysis

In this subsection, comparative experiments are conducted to test the ability of the proposed GLPSVM. We compare it with SVM, SVM+LDA, MCVSVM, and MCLPV_SVM on seven different databases selected from the well-known UCI database. The seven databases include three binary databases and four multiclass databases. In the multiclass tasks, one-against-one coding strategy is employed. The detailed information of these selected databases is shown in Table 3. For all these databases, 30% of samples in each class are randomly selected for training, the remaining samples are used for testing, and all the data samples are normalized before experiment.

Table 4 gives the classification accuracy of SVM, SVM+LDA, MCVSVM, MCLPV_SVM, and GLPSVM under different regularization parameter, neighborhood parameter, and heat kernel parameter, where the highest average classification accuracy is presented. Here, the average classification accuracy is obtained through repeating the operation 20 times. It can be seen from Table 4 that the proposed GLPSVM has always the highest accuracy, especially on the Wine and the Iris databases. The accuracy is more than 97%, far more than the other algorithms.

## 5. Conclusions

In this paper, a new extension of SVM was proposed, which was called support vector machine with globality-locality preserving (GLPSVM). It took the intrinsic manifold structure of the data space into consideration. Besides, the soft margin GLPSVM was also presented. The effective algorithm of GLPSVM showed that the model could be solved through transferring it to the standard SVM model and using the standard SVM software package for solving, which would greatly improve the implementation efficiency. Finally, experimental results on real world databases validated that the proposed method could have better performance than SVM, SVM+LDA, MCVSVM, and MCLPV_SVM.

## Figures and Tables

**Table 1:** The effects of parameter *C* and sample neighborhood *k*_1_ on classification performance.

*k*_1_	10	20	30	40	50	60	70	80
*C* = 0.1	0.8207	0.8048	0.8008	0.8008	0.7888	0.7769	0.7888	0.7809
σ =	2	1	2	0.5	0.5	2	1.5	2

*C* = 1	0.8884	0.8884	0.8964	0.8805	0.8765	0.8008	0.8207	0.8048
σ =	0.5	0.5	0.5	0.5	0.5	0.5	0.5	0.5

*C* = 10	0.9323	0.9482	0.9044	0.9203	0.9323	0.9203	0.9084	0.8884
σ =	2	0.5	2.5	0.5	2.5	1	0.5	0.5

*C* = 100	0.9402	0.9283	0.9402	0.9442	0.9402	0.9323	0.9482	0.9363
σ =	1.5	2	1.5	1.5	2.5	0.5	1	0.5

**Table 2:** The effects of trade-off parameter λ on classification performance; let σ_1_ = σ_2_ = 2.

λ	0.2	0.4	0.8	1	1.2	1.4	1.6	2
*C* = 0.1	0.8048	0.8048	0.7849	0.8008	0.8088	0.7928	0.7928	0.8008
*k*_1_	40	30	30	10	50	20	90	10

*C* = 1	0.9441	0.7849	0.8167	0.8127	0.7968	0.7968	0.8008	0.8008
*k*_1_	10	10	20	20	10	30	10	10

*C* = 10	0.9203	0.8964	0.9004	0.9203	0.8845	0.8964	0.8884	0.8645
*k*_1_	30	30	10	20	10	10	10	10

*C* = 100	0.9283	0.9442	0.9243	0.9482	0.9203	0.9323	0.9283	0.9163
*k*_1_	50	20	20	20	30	20	20	10

**Table 3:** Database information for comparative analysis.

Database	Number of sample	Attribute	Number of class
Breast	699	9	2
Heart	270	13	2
Pima	768	8	2
New Thyroid (NT)	215	5	3
Wine	178	13	3
Iris	150	4	3
Glass	214	9	6

**Table 4:** Classification accuracy for comparative analysis.

Database	SVM	SVM + LDA	MCVSVM	MCLPV_SVM	GLPSVM
Breast	0.8613 ± 0.0103	0.9572 ± 0.0063	0.9596 ± 0.0052	0.9658 ± 0.0045	0.9686 ± 0.0046
*C* = 1				*k* = 40	*k*_1_ = 40, *k*_2_ = 1
				*t* = 2	σ_1_ = σ_2_ = 2

Heart	0.6912 ± 0.0352	0.8176 ± 0.0205	0.8235 ± 0.0186	0.8294 ± 0.0338	0.8471 ± 0.0243
*C* = 10				*k* = 50	*k*_1_ = 50, *k*_2_ = 1
				*t* = 2	σ_1_ = σ_2_ = 2

Pima	0.6723 ± 0.0296	0.7689 ± 0.0158	0.7663 ± 0.0144	0.7689 ± 0.1023	0.7841 ± 0.0277
*C* = 10				*k* = 80	*k*_1_ = 80, *k*_2_ = 1
				*t* = 2	σ_1_ = σ_2_ = 2

NT	0.6923 ± 0.0000	0.8965 ± 0.0157	0.8569 ± 0.0148	0.9496 ± 0.0144	0.9512 ± 0.0170
*C* = 100				*k* = 10	*k*_1_ = 10, *k*_2_ = 2
				*t* = 0.5	σ_1_ = σ_2_ = 0.5

Wine	0.5714 ± 0.0275	0.9490 ± 0.0228	0.8163 ± 0.0126	0.9694 ± 0.0219	0.9796 ± 0.0207
*C* = 100				*k* = 20	*k*_1_ = 20, *k*_2_ = 2
				*t* = 2	σ_1_ = σ_2_ = 2

Iris	0.9467 ± 0.0172	0.9713 ± 0.0139	0.9733 ± 0.0147	0.9333 ± 0.0140	0.9753 ± 0.0124
*C* = 1				*k* = 10	*k*_1_ = 10, *k*_2_ = 2
				*t* = 0.5	σ_1_ = σ_2_ = 0.5

Glass	0.6508 ± 0.0179	0.7996 ± 0.0132	0.7967 ± 0.0098	0.9172 ± 0.2222	0.9402 ± 0.0219
*C* = 100				*k* = 4	*k*_1_ = 4, *k*_2_ = 4
				*t* = 0.5	σ_1_ = σ_2_ = 0.5
